# Settlement of Physicians’ Accounts

**Published:** 1867-02

**Authors:** 


Settlement of Physician's Accounts.—The Secretary of War orders that paragraph 1,308, Revised Regulations of the Army, be modified so that a physician's account of pay due, in the ordinary form of an officer's pay account, shall be presented to a paymaster for payment, vouched for by a certificate thereon by the commanding officer that it is correct and agreeably to contract, and that the services have been duly rendered, which certificate he will not make unless the contract has been approved by the Surgeon-General or the Medical Director of the Department.

